# Retraction: *De Novo* Transcriptomic Analysis of an Oleaginous Microalga: Pathway Description and Gene Discovery for Production of Next-Generation Biofuels

**DOI:** 10.1371/annotation/3155a3e9-5fbe-435c-a07a-e9a4846ec0b6

**Published:** 2012-06-29

**Authors:** LingLin Wan, Juan Han, Min Sang, AiFen Li, Hong Wu, ShunJi Yin, ChengWu Zhang

It has been brought to the attention of the PLoS ONE Editors that a substantial part of the text in this article was appropriated from text in previous publications, including the articles below:

Transcriptome sequencing and annotation of the microalgae Dunaliella tertiolecta: pathway description and gene discovery for production of next-generation biofuels.

BMC Genomics. 2011 Mar 14;12:148.

Deep sequencing of the Camellia sinensis transcriptome revealed candidate genes for major metabolic pathways of tea-specific compounds.

BMC Genomics. 2011 Feb 28;12:131.

An efficient approach to finding Siraitia grosvenorii triterpene biosynthetic genes by RNA-seq and digital gene expression analysis.

BMC Genomics. 2011 Jul 5;12:343.

Examination of triacylglycerol biosynthetic pathways via de novo transcriptomic and proteomic analyses in an unsequenced microalga.

PLoS One. 2011;6(10):e25851.

PLoS ONE therefore retracts this article due to the identified case of plagiarism.

